# Long COVID: Long-Term Impact of SARS-CoV2

**DOI:** 10.3390/diagnostics14070711

**Published:** 2024-03-28

**Authors:** Huda Makhluf, Henry Madany, Kenneth Kim

**Affiliations:** 1 Department of Mathematics and Natural Sciences, National University, San Diego, CA 92123, USA; 2 Center for Infectious Disease, La Jolla Institute, La Jolla, CA 92037, USA; hmadany@lji.org (H.M.); kenneth@lji.org (K.K.); 3 Public Health Sciences, University of California, Irvine, CA 92697, USA

**Keywords:** long COVID (LC), SARS-CoV-2, chronic fatigue syndrome, post-acute sequelae of SARS-CoV-2 (PASC), autoimmunity, machine learning, artificial intelligence, precision

## Abstract

Four years post-pandemic, SARS-CoV-2 continues to affect many lives across the globe. An estimated 65 million people suffer from long COVID, a term used to encapsulate the post-acute sequelae of SARS-CoV-2 infections that affect multiple organ systems. Known symptoms include chronic fatigue syndrome, brain fog, cardiovascular issues, autoimmunity, dysautonomia, and clotting due to inflammation. Herein, we review long COVID symptoms, the proposed theories behind the pathology, diagnostics, treatments, and the clinical trials underway to explore treatments for viral persistence, autonomic and cognitive dysfunctions, sleep disturbances, fatigue, and exercise intolerance.

## 1. Introduction

A new crisis is emerging in the wake of the COVID-19 pandemic, with 30 to 60% of COVID-19 patients developing long COVID (LC). LC has been given several names: post-acute SARS-CoV-2 sequelae (PASC), post-acute COVID-19 syndrome (PACS), post-COVID-19 condition (PCC), among others. People suffering from LC are called “long haulers”, in reference to the condition’s persistent, lingering symptoms that prevent COVID-19 survivors from returning to their pre-infection selves within 12 weeks of the illness.

The most common symptoms of LC are extreme fatigue, shortness of breath, loss of smell, and muscle aches. LC, however, affects almost every organ system in the human body and manifests in over two hundred symptoms. According to the most recent definition by the World Health Organization, LC can affect anyone who was exposed to SARS-CoV-2 regardless of the severity of the infection and symptoms. The CDC cites the interim federal working definition by the National Research Action Plan on LC (Chapter 1, p 14) as follows: “*Long COVID is broadly defined as signs, symptoms, and conditions that continue or develop after initial COVID-19 or SARS-CoV-2 infection. The signs, symptoms, and conditions are present four weeks or more after the initial phase of infection; may be multisystemic; and may present with a relapsing–remitting pattern and progression or worsening over time, with the possibility of severe and life-threatening events even months or years after infection. LC is not one condition. It represents many potentially overlapping entities, likely with different biological causes and different sets of risk factors and outcomes*”. 

The underlying biological causes of LC are still unknown, but there are many proposed theories under investigation: (1) viral persistence in nerve tissues and other tissues in the body, (2) dysfunction of the immune system that persists post-infection, (3) reactivation of dormant viruses, such as the Epstein–Barr Virus (EBV), (4) impairment of blood flow due to endothelial cell damage, and (5) genetic predispositions leading to production of autoantibodies against type I interferons [1]. These hypotheses are not mutually exclusive and may be sequential; for example, a lingering viral presence can cause chronic inflammation, triggering blood clot formation. 

LC could be driven by a persistent viral infection, by a reactivation of latent herpes viruses such as EBV and varicella Zoster virus, inflammation, autoimmune processes, and hypercoagulation. When compared to people who either had recovered from COVID or had never contracted the virus or developed the disease, people with LC, were found to have lower levels of cortisol [2] and an elevated level of antibody responses to spike protein. This would suggest a persistent presence of viral antigens stimulating the production of these antibodies. 

Beyond the immune system, LC affects most organs and organ systems in the body, including the heart [3,4,5], lungs, brain [6,7,8,9,10], kidneys [11,12,13], liver, and gut in general [14,15,16], including the spleen, pancreas [17,18,19,20,21,22], and also the reproductive system [23,24]. Resulting symptoms include chronic fatigue, shortness of breath, postural orthostatic tachycardia syndrome (POTS), palpitations, myalgias, arthralgias, cognitive impairment, headaches, sleep disturbances, secondary anxiety, depression, nausea, and diarrhea. Other symptoms may be grouped under myalgic encephalomyelitis/chronic fatigue syndrome (ME/CFS) [25]. Other notable symptoms such as hair loss, loss of smell and taste, and low sperm counts, are observed in LC. Referring to prior ME/CFS findings may help direct LC research toward more effective therapies. Currently, there is no effective treatment for ME/CFS.

## 2. Multiorgan Symptom Involvement in LC

Out of several hundred million cases worldwide, more than one million people in the United States and at least 6 million worldwide have died due to COVID-19 (Johns Hopkins Coronavirus Resource Center). Many survivors suffer from multiorgan symptoms in the lungs, heart, gut, kidneys, liver, and brain, all of which have been attributed to LC. 

### 2.1. Cardiovascular and Gastrointestinal

Using the national healthcare databases from the US Department of Veterans Affairs, Xie et al. (2022) studied cardiovascular manifestations of LC in order to estimate the risks of cardiovascular outcomes in COVID-19 survivors. The increased risk of incident cardiovascular disease included heart failure, myocarditis, pericarditis, dysrhythmias, non-ischemic and ischemic heart disease, and thromboembolic disease, even for individuals who were not hospitalized during their acute COVID-19 infection phase [26]. The same team used those same databases to evaluate the risks and burdens of gastrointestinal disorders in LC and reported increases in disorders such as peptic ulcer disease, pancreatitis, hepatic and biliary disease, motility disorders, dyspepsia with stomach pains, indigestion, bloating, and reflux [27]. Remarkably, studies investigating the gut microbiota composition of LC patients found lower levels of *Faecalibacterium prausnitzii* and higher levels of *Bacteroides vulgatus* and *Ruminococcus gnavus*. Healthful butyrate-producing bacteria such as *Bifidobacterium pseudocatenulatum* and *Faecalibacterium prausnitzii* showed an inverse correlation with LC at 6 months [28], demonstrating the negative effect of LC on the gut microbiome.

### 2.2. Neurological Impairment

Neurological impairment, a hallmark of LC, includes brain fog, memory loss, loss of taste and smell, dizziness, numbness and tingling, and autonomic dysfunctions [24,29]. Fernández-Castañeda found striking similarities between the neuropathophysiological and cognitive impairments following cancer treatment and LC—typically driven by neuroinflammation and microglial reactivity which leads to dysregulation of neurogenesis in the hippocampus [30]. Human subjects suffering from cognitive impairment and deficits due to LC further demonstrated elevated CCL11 in the plasma compared to the control groups [30]. 

Fernández-Castañeda and colleagues used a mouse model to investigate the underlying mechanisms behind neuroinflammation caused by elevated levels of CCL11. They found that cognitive impairment in mice was linked to microglial activation combined with loss of myelination and inhibition of neurogenesis. Microglia are resident macrophages in the central nervous system that play a role in homeostasis and refinement of neuronal networks. In the hippocampus, activation of microglia created a neurotoxic state and inhibited neurogenesis, potentially leading to impaired memory formation. Persistently elevated levels of CCL11 appear to be the culprit, and systemic injection of CCL11 into mice went on to cause the activation of hippocampal microglia. These findings are consistent with results from a cohort of humans with brain fog. They offer promising insights and shed light on CCL11 and microglial modulation as a potential therapeutic intervention. Given the small sample size and strain-specificity (Wuhan-Hu-1 isolate or USA-WA1/2020) used in this study, the findings warrant robust future testing with a larger cohort of patients. Additionally, other cell types may be substantially contributing to brain fog, such as astrocytes, as noted by Fernández-Castañeda and colleagues, and will necessitate further investigation. 

### 2.3. Dysautonomia and Myalgic Encephalomyelitis and Chronic Fatigue Syndrome

Dysautonomia and symptoms related to Myalgic Encephalomyelitis and Chronic Fatigue Syndrome (ME/CFS) overlap with the clinical manifestations of LC. Common symptoms and include persistent fatigue, post-exertional malaise, postural orthostatic tachycardia syndrome (POTS), disrupted sleep, cognitive issues, mitochondrial fragmentation, myalgias, and arthralgias [25]. While the onset of ME/CFS has been associated with EBV, Q fever, and influenza, there is currently insufficient evidence to classify SARS-CoV-2 as another infectious trigger in the ME/CFS [31]. Bonilla et al. collected retrospective data from 140 patients with LC, using the Institute of Medicine 2015 criteria to determine the ME/CFS phenotype, and found that about 43% of the LC patients had the ME/CFS subtype. Most of the patients in the cohort, however, were female subjects in an affluent area, with little representation of minority populations. A larger and more diverse population is needed to substantiate these findings [32]. Interestingly, cyclical variations in menstrual symptoms have also been recognized in female ME/CFS and LC patients [33].

### 2.4. Immune Dysregulation

Immune deregulation with inflammation and persistent immune activation was observed with LC. Klein et al. identified key immunological manifestations for LC, using specific circulating myeloid and lymphocyte populations compared to matched controls. There were increases in (1) non-classical monocytes, typically associated with anti-inflammatory responses, (2) activated B cells, (3) double-negative B cells, (4) exhausted T cells, and (5) IL4/IL6 secreting T cells, which correlated with antibody reactivity mounted against Epstein–Barr Virus antigens. Remarkably, LC patients showed significant decreases in cortisol levels compared to matched control groups, and also demonstrated significant decreases in circulating type I dendritic cells (DC1) subsets, which typically promote cytotoxic CD8+ differentiation [2].

Phetsouphanh et al. reported immunological dysfunction that persisted for eight months in LC patients with activated innate immune cells and elevated expression of type I IFN (IFN-β) and type III IFN (IFN-λ1) [34]. Other studies pointed out a triad of elevated cytokines, including IL-1ß, IL-6, and TNF-α, all of which remained deregulated in LC [35].

Viral persistence in tissues may be driving LC. Tejerina et al. reported the detection of SARS-CoV-2 RNA in plasma, stool, and urine in patients with persistent symptoms after COVID-19 [36]. Other studies established the presence of residual virus in breast tissue and the appendix and suggested that the gastrointestinal tract is acting as a viral reservoir [27,28]. Ceulemans et al. reported the persistence of SARS-CoV-2 RNA in lung tissue after mild COVID-19 [37], and other studies reported the detection of residual SARS-CoV-2 viral antigens in GI and hepatic tissues from five recovered patients with COVID-19 [38].

## 3. Children and LC 

Whether or not children can and do get LC has long been discussed in scientific circles. The occurrence of other post-viral syndromes in children, e.g., those after CMV and Epstein–Barr virus infections, in addition to Guillain–Barre, and the better-understood though rare MIS-C complicating a COVID-19 infection, would all support the likelihood that children too can be affected by long COVID. Another ongoing debate has been whether the persistent symptoms attributed to LC are directly caused by the infection or only indirectly so, e.g., time away from school, increased stress, and social distancing. 

A Danish study followed more than 10,000 children 0 to 14 years of age with confirmed SARS-CoV-2 infection. Persistent symptoms at two months were found to be higher in all ages compared with a matched control group (0 to 3 years: 40.0% vs. 27.2%; 4 to 11 years of age: 38.1% vs. 33.7%; 12 to 14 years: 46.0% vs. 41.3%) [39]. 

Nevertheless, the prevalence of long-term COVID-19 in children appears to be lower than in adults, with a prevalence of 4% or less [40,41,42]. Risk factors include older age, female sex, allergic diseases, and poorer pre-COVID health [39,41,43,44,45]. 

Another study from Scandinavia, this time at the University of Gothenburg, Sweden, analyzed 162,383 children aged 6–17 years over a two-month period and then reevaluated them 9 months later. Children in the highest-risk group for LC included (1) girls (an incidence of 0.19 vs. 0.12 in boys per 100 person-years), (2) youth (12–17 years old), and (3) those with chronic conditions or previous hospitalization. The incidence of long-term COVID was similar across parental education levels but was six times higher if parents had also had long-term COVID-19. 

Dun-Dery et al. investigated the proportion of children with post-COVID-19 symptoms at 6- and 12-months following SARS-CoV-2 testing at 14 Canadian pediatric emergency departments as members of the Pediatric Emergency Research Canada network [46]. At 6 months, 5147 children (1152 with SARS-CoV-2 positive tests and 3995 with negative tests) were followed up. LC symptoms and quality of life changes were observed in 6 of 1152 children with positive SARS-CoV-2 tests (0.52%) and 4 of 3995 children with negative SARS-CoV-2 tests. At 12 months out, 5563 children (1192 with SARS-CoV-2 positive tests and 4371 with negative tests) were tracked for LC symptoms. This time, LC symptoms and quality of life changes were observed in 8 of 1192 children with positive SARS-CoV-2 tests and 7 of 4371 children with negative SARS-CoV-2 tests. In the first group, children reported neurologic, respiratory, and systemic symptoms. Notably, in this Canadian study, few of these children who developed LC suffered from a low quality of life [46]. 

In another prospective observational cohort study, Haddad et al. investigated LC symptoms in both exposed and infected children, adolescents, and their parents one year after SARS-CoV-2 infection in Germany. A total of 341 households with 723 adults, 140 adolescents aged 14 to 18 years, and 404 children less than 14 years of age, were classified as having household exposure to SARS-CoV-2 without infection or SARS-CoV-2 infection. Participants were assessed for LC symptoms by completing an online questionnaire 11 to 12 months post-infection. Infected adolescent girls and infected women and men were found to be at an increased risk of negative outcomes and seemed to be clustered within families. LC interventions at the family level were thus seen to prove beneficial [47]. 

Zheng et al. conducted a meta-analysis to review the prevalence and risk factors for LC in children and adolescents. The findings suggested that about one-quarter of pediatric patients had LC symptoms involving multi-organ systems. These were based on 40 studies totaling 12,424 participants [48]. They also reported, as have other studies, that females were more likely to experience LC and again found that factors such as older age and poor mental or physical status were associated with LC in children and higher in adolescents compared to younger children [48]. 

Management of children with long COVID continues to evolve according to protocols that (1) are based on expert opinions, (2) are extrapolated from guidelines in adult medicine, and (3) rely on already established approaches used to manage other chronic pediatric conditions like chronic fatigue syndrome and migraine headaches. Parent involvement is crucial in managing the stress of a child with long-term COVID-19. Given a child’s limited ability to communicate what they feel, a perceptive parent can pick up more subtle signs of distress, e.g., frustration at being unable to complete once routine tasks, symptoms of dysautonomia, and somatization signs, e.g., headaches, fatigue, and nausea. An integrated approach that combines physical and mental health is clearly indicated. 

It is noteworthy that research studies relying on surveys and questionnaires can pose certain challenges. The reliability of control groups is a key factor to be taken into consideration, particularly, given that the majority of SARS-CoV-2 infections have been asymptomatic or pauci-symptomatic in children, and especially so during the early prototypical and variants outbreaks.

## 4. Diagnostics and Treatment

Many manifestations of LC are not measurable using clinical testing, making a clinical history an important first step in the diagnosis of LC. LC is a diagnosis of exclusion [49]; thus, known causes of specific symptoms must be ruled out prior to the establishment of LC as a diagnosis. Because LC manifests across multiple organ systems, differential diagnoses will vary based on symptoms. For example, in addition to standard blood and urine testing, patients demonstrating cardiovascular symptoms will receive ECG, echocardiogram, and other cardiovascular evaluations whereas those with neuropsychiatric manifestations will be evaluated using EEG, MRI, and other neurologic tests prior to symptoms being attributed to LC. 

### 4.1. Testing-Defined Clusters of LC

Very recently, data from the NIH N3C (National Cohort Collaborative) and RECOVER (REsearching COVID to Enhance Recovery) programs were utilized to identify six LC phenotype clusters in over 6000 LC patients using machine learning [50]. Only one cluster (designated Multisystem + lab) largely exhibited laboratory abnormalities of hyperglycemia, hypocalcemia, elevated serum creatinine, elevated AST, elevated ALP, elevated ferritin, lymphopenia, and thrombocytopenia. This cluster was also associated with a worse prognosis. A second cluster (designated pulmonary) exhibited hypoxemia without cardiac palpitations. Two separate clusters (designated cardiovascular and Multisystem + pain) showed palpitations in addition to hypoxemia. Thus following a diagnosis of LC through exclusion of other differentials, a review of the following tests may assist in subphenotyping LC for the purpose of guiding treatment: CBC, serum chemistry, oximetry, and ECG.

A commitment to artificial intelligence, machine learning, and computational science may help to solve the LC problem. LC is challenging both in its diagnosis and treatment, given its heterogeneous, non-monolithic symptoms, coupled with diverse sets of risk factors and outcomes. Leveraging technology and harnessing the power of AI to analyze vast datasets and surface insights on biomarker development, prognosis predictions, and tailored treatment may all bolster genuine efforts toward a full-LC recovery.

Such a machine learning algorithm was deployed to characterize data from patients with LC using ICD-10 diagnostic code U09.9 as a primary code applied to electronic medical records. Despite the challenges posed by the variation in the quality of electronic medical records, the algorithm identified different LC subtypes with neurological, gastrointestinal, respiratory, and cardiopulmonary clustering features [51] (Table 1). Remarkably, Pfaff et al. discovered that patients who had received the U0.9.9 code were primarily female, White, non-Hispanic individuals living in areas with low unemployment and poverty, possibly highlighting disparities in the LC diagnosis. The sample size was small (*n* = 33,782), and EHR data could have been limited to patients with access to healthcare, while patients with barriers to healthcare would have been underrepresented in the dataset. Further research is needed for the development and validation of these clusters [51].

Using the N3C EHR data repository, XGBoost (Extreme Gradient Boosting), a powerful and flexible machine learning algorithm, was used to identify who has LC in the USA [52]. Data from 597 LC patients, regardless of their hospitalization status, were used to train three machine-learning models to identify LC patients amongst COVID-19 patients. These models identified potential LC patients with high precision. LC clinical manifestations are complex, involving a wide range of multiorgan involvement, rendering the sample size crucial when mining various databases. With an increasing number of patients with LC and the accumulation of more data points, these models can be refined and retrained to evolve the algorithms with additional emergent evidence to solve the LC problem [52].

Baseline chronic inflammation may be considered a key contributor to LC symptoms and the diversity in its clinical manifestations. Understanding it could provide hope and optimism for the potential treatment of LC and tackling its associated challenges.

Using two British birth cohorts, the 1958 National Child Development Study (NCDS) and the 1970 British Cohort Study (BCS70), to allow comparison between generations, Staatz et al. assessed the risk of severe COVID-19 and LC in relation to the age at which the participant first became obese or overweight compared to participants whose weight had remained healthy throughout their lives. They found that obesity may be considered a state of chronic inflammation and that an elevated BMI throughout life increases the risk of various diseases, including LC [53]. Additionally, they showed that there is an increased risk of LC for individuals who first experienced obesity at a younger age compared to those who were never obese [53].

Combining scRNA-seq, mass cytometry, and scATAC-seq to compare immune cell types in peripheral blood collected from young and old subjects and patients with COVID-19, Zheng et al. found that COVID-19 promoted age-induced immune cell polarization and gene expression related to inflammation and cellular senescence [54]. Additionally, age-associated dendritic cells were found to increase IFN-stimulated gene expression and decrease antigen-presenting ability [54].

Krishna et al. investigated the levels of IFN-γ from PBMCs of patients with LC and from patients who had recovered from acute COVID-19 infection [55].

*54 unexposed donor samples were recruited by the National Institute for Health Research BioResource Cambridge through the Anti-viral Responses in Ageing study before October 2019, so these patients were not exposed to SARS-CoV-2. 55 LC patients were recruited for the study based on LC symptoms that persisted for at least five months after acute COVID-19* [55].

Persistently high levels of IFN-γ from PBMCs of patients with long COVID compared to the PBMCs from patients recovering from acute SARS-CoV-2 infection without ex vivo peptide stimulation were detected using sensitive Fluorospot assays. This IFN-γ release was mediated using CD8 positive T cells and dependent on antigen presentation of 14 positive cells. No scRNA sequencing data, however, were performed to analyze this population of CD8+ T cells in patients with long COVID. This could have helped to characterize the activation profiles of these cells more fully [55]. A considerable number of the LC patients had symptoms resolve spontaneously or after vaccination. There was a significant decrease in interferon-gamma after vaccination. Interestingly, a reduction in IFN-γ production to baseline levels correlated with improvement and resolution of LC symptoms. The authors concluded that, at least at this stage, whether the interferon-gamma is a mediator, or a potential biomarker of LC is still unclear.

### 4.2. Therapeutics

Treatment of LC addresses both viral and extraviral etiologies. Following a diagnosis of LC, treatment is based on symptomology, and laboratory and clinical findings [56,57]. Given that many people with LC have symptoms affecting their nervous, mental, respiratory, and cardiovascular systems, a team of specialists in cardiology, pulmonology, neurology, and mental health may be deployed to deliver tailored treatment for an individual’s syndrome. Several therapeutics are being investigated for the treatment of LC (Table 2). Antiviral drugs include ritonavir/Nirmatrelvir (Paxlovid), a viral protease inhibitor, and Remdesivir and Favipiravir, RNA-dependent RNA polymerase inhibitors. A remarkable study by Wong and colleagues [58] found that serotonin levels were reduced in people with LC, and that restoring the levels of circulating serotonin might improve LC symptoms. Anti-inflammatory therapies are also under investigation, and these include, Imatinib (kinase inhibitor), Infliximab, (TNF-α inhibitor), and Ibudilast (a phosphodiesterase inhibitor). Scheppke et al. reported on three cases of highly functioning individuals who suffered severe fatigue and cognitive impairment due to LC and remarkably experienced rapid remission following a casirivimab/imdevimab cocktail of monoclonal antibody infusions irrespective of their age, sex, or vaccination status. These reports suggest that the monoclonal antibody cocktail treatment may be effective against LC. The authors suggest that this rapid remission could provide mechanistic implications for managing and possibly treating other post-viral chronic conditions [59].

### 4.3. Biomarkers for Severe Cognitive Slowing

Two short web-based cognitive tasks were administered to patients with LC to examine cognitive slowing using Simple Reaction Time and Number Vigilance Tests. At two clinics in Germany and the UK, patients with LC were age-matched to healthy individuals who contracted SARS-CoV2, recovered without manifesting LC, and received a self-administered psychomotor assessment on their laptops [65,66]. Patients with LC showed about three standard deviations slower reaction times compared to the control. This thirty-second psychomotor task could be beneficial as a diagnostic work-up and biomarker to establish a baseline measurement and track the progress and improvements following rehabilitation. 

### 4.4. Oral Bacteriotherapy and Probiotics

Several studies have suggested that probiotics and bacteriotherapy may be effective remedies for managing LC. Probiotics and synbiotics may improve some symptoms of LC, specifically fatigue, memory loss, and gastrointestinal upset; however, clinical data and evidence are still limited (Table 3). In the randomized placebo-controlled and double-blind clinical trial, patients with long COVID received either a placebo (*n* = 231) twice daily for six months or a symbiotic preparation, SIM01, (*n* = 232) characterized by an encapsulated lyophilized bacterial powder containing B adolescentis, Bifidobacterium bifidum, and Bifidobacterium longum with prebiotic compounds including galacto- and xylo-oligosaccharides and dextrin [67]. 

Lau and colleagues found that SIM01 treatment alleviated the neuropsychiatric symptoms of LC, including concentration difficulties and memory loss. The precise mechanisms underlying these improvements warrant further investigation. It could be linked to the lack of gut bacteria that produce short-chain fatty acids, which help regulate the immune response. A follow-up study, designed as a single-center, triple-blind, randomized, placebo-controlled clinical trial, will aim at evaluating the effectiveness of modifying gut microbiota, boosting immunity, and reducing long-term complications and co-morbidities in post-COVID conditions. The emphasis will be on outcomes within a six-month period, comprising cardiopulmonary complications and neuropsychiatric disorders. 

The efficacy of Lactobacillus Paracasei PS23 for LC Patients was researched (NCT05813899 [68]). Researchers will evaluate whether probiotics PS23 improve patients’ symptoms by lowering blood cortisol and inflammation-related indicators. 

Another clinical study aims to understand the effects of supplementing an orally ingested Lactobacillus plantarum 299v, Lp299v, on SARS-CoV-2-associated endothelial dysfunction via the reduction in cf-mtDNA, TLR9 activation, and inflammation. Malik and colleagues showed that Lactobacillus Plantarum 299v supplementation improved vascular endothelial function and reduced inflammatory biomarkers in men with stable coronary artery disease [69]. They will investigate (NCT05227170 [70]) the impact of Lp299v on vascular function in LC patients. 

(NCT04813718 [71]) will evaluate a dietary supplement, Omni-Biotic Pro Vi 5, to elucidate the mechanisms for post-COVID pulmonary fibrosis and the gut–lung axis. The study will measure the microbiome composition using 16 sRNA sequencing and changes in TNF alpha, IL-1b, IL-6, IL-6R, IL-8, IL-10, IL-17, and IL-23 levels of serum over time and with or without the intervention. 

**Table 3:** Selected oral bacteriotherapy and probiotics for LC treatment.

Clinical Trial Identifier	Treatment	Outcomes
NCT05813899 ^7^	Lactobacillus Paracasei PS23	Cortisol and Inflammation indicators
NCT05227170 ^8^	Lactobacillus plantarum 299v	Vascular Endothelial Function
NCT04813718 ^9^	Omni-Biotic Pro Vi 5	Microbiome Composition and Inflammation Indicators

^7^ [68] ^8^ [70] ^9^ [71].

## 5. Mouse Models

Mouse models are invaluable for research and the development of new effective therapies and drugs. Modeling human pathologies observed in COVID-19 and LC using complex humanized mouse models will give scientists a clear window into investigating various therapeutics. Prolonged infections of mouse models with SARS-CoV-2 for an extended period may also provide valuable insights into LC development. Just recently, Dos Santos Alves and colleagues showed that human coronavirus OC43-elicited CD4+ T cells are protective against SARS-CoV-2 in HLA transgenic mice [72]. This validates that SARS-CoV-2-reactive T cells are detected in some healthy, unexposed individuals, just as other human studies indicate these T cells could be elicited by the common cold coronavirus OC43. Investing in animal models that can accurately recapitulate the range of LC symptoms is paramount. This will allow scientists to investigate the underlying molecular and cellular mechanisms of how SARS-CoV2 could lead to chronic LC disease. Animal models will also be valuable in screening potential therapeutics. 

## 6. RECOVER Clinical Trials

Researching COVID to Enhance Recovery (RECOVER) is a project funded by the National Institute for Health to research LC. It uses an innovative platform design and protocol that allows for randomized controlled trial data collection across multiple sites. It allows for more efficient strategies to surface insights quickly by enrolling, testing, and introducing new interventions in a coordinated fashion. As of August 2023, two clinical trials are listed, RECOVER-VITAL and RECOVER-NEURO, to evaluate interventions for viral persistence, reactivation, immune dysfunction, and neurocognitive dysfunctions, respectively. Two additional protocols for RECOVER-Autonomic and RECOVER-Sleep are still pending (RECOVER). Clinical Trial Registry ID NCT05965726 [73] aims to determine the effects of Paxolovid in two dosing durations, 15 days, and 25 days, for the treatment of LC and test the hypothesis that antiviral therapies may result in viral clearance and reduced inflammation. NCT05965752 [74] aims to test interventions that could potentially ameliorate deficits and declines in cognition, executive function, and attention, using cognitively stimulating activities like puzzles and games that engage and adaptively challenge participants. BrainHQ, a groundbreaking brain training program that leverages the principles of neuroplasticity, will be used to gauge improvements in processing speed, attention, and memory. Another intervention will deploy Transcranial direct current stimulation (tDCS), a non-invasive and safe brain stimulation technique. It will be tested using a home-based device that delivers a weak electrical current to target the dorsolateral prefrontal cortex region of the brain (RECOVER).

## 7. Discussion

There is no one-size-fits-all approach to LC. Many factors complicate the understanding and treatment of LC. These include genetic predispositions, prior infections with EBV or other herpes viruses, individual BMI differences, microbiome composition, baseline inflammation, gender, and age, fitness advantage, viral load, coupled with the uniqueness of the evolving viral variant with the corresponding amino acid changes and the prolonged stage between exposure and LC onset. A treatment regimen, including drugs, that could be helpful to one patient might cause adverse effects in another.

Metformin, a drug used to treat type 2 diabetes and control the amount of glucose in the blood, was tested in a randomized, placebo-controlled, phase 3 trial to investigate the effect of outpatient COVID-19 treatment on the incidence of LC. In total, 1126 participants were randomly assigned to a placebo group or an experimental group receiving 500 mg metformin pills, one on the first day, twice daily for four days, followed by 500 mg in the morning and 1000 mg in the evening for nine days. Metformin was found to reduce by 42% the LC incidence and was thought to do so in a safe, cost-effective manner with minimal drug interactions and contraindications [75]. The benefit of metformin and its potential impact on reducing oxidative stress and inflammation warrants future research to reproduce the results, confirm the findings, and implement metformin as a COVID-19 treatment to prevent LC.

Ma and colleagues investigated the effects of caloric restriction on the aging process in a rat animal model by building a single-cell transcriptomic atlas with a particular focus on immune cells. The findings revealed that excessive inflammation can be reversed by caloric restriction [76]. In humans, prolonged caloric restriction decreases fasting insulin levels and body temperature [77]. Long-term fasting was thus considered to be an effective intervention for managing and treating LC [78], albeit in a very small study of LC patients. Nutritional ketosis will ensue when the body switches to burning fat as a primary energy source. Luda et al. showed that ketone bodies (KBs)-including β-hydroxybutyrate (βOHB) and acetoacetate (AcAc) are essential fuels in supporting CD8+ T cell metabolism and effector function [79], and that ketolysis is a metabolic and epigenetic driver of optimal CD8+ T cell effector responses. The authors acknowledge that these experiments were performed with murine T cells. Thus, the extent to which these findings can be extrapolated to humans remains to be elucidated [79]. Nonetheless, nutritional approaches that lead to a decrease in inflammation and oxidative stress may be beneficial.

### 7.1. Nutrition, Diabetes, and Health Outcomes

During the 2020 global pandemic, SARS-CoV-2 disproportionately affected racial minorities in the US. Significant disparities were documented primarily among Latino individuals and African Americans, with higher mortality rates than white individuals. This could include the frequency of type II diabetes as an underlying comorbid health condition. The gut microbiome plays a significant role in the pathogenesis of obesity and obesity-related metabolic dysfunction [80]. Unhealthy diets could change the composition of the microbiome, causing an increase in inflammation and resulting in the glucose intolerance and insulin resistance typically observed in type II diabetes. In a recent study, Wang et al. demonstrated that the association between a healthful Mediterranean diet and the prevalence of diabetes in US Latino populations is strongly modified by the profile of an individual’s gut microbiome. Loss of species diversity and disturbances in the composition of a gut microbiome could result in dire consequences on human health [81]. While further research is needed to elucidate the underlying mechanisms between nutrition and disease, adherence to a Mediterranean diet may help improve long-term health outcomes regarding LC comorbidities, serving as a possible starting point in addressing the weight of racial disparities in LC health outcomes. 

There is a connection between comorbidities and LC. Falsetti and colleagues established a strong correlation between comorbidities and the subsequent development of LC [82]. Their findings stress that patients with hypertension, obesity, diabetes, chronic lung disease, and heart disease are dramatically impacted by COVID-19 and are more likely to develop LC [82]. Possible explanations for such associations included an exacerbated immune response and immune dysfunction, endothelial dysfunction, and acute illness. The authors caution that additional research is needed to gain a precise understanding of LC and comorbidities. They emphasize the complexity of LC and the significant inter-individual variation, as well as the viral genotype at play [82].

### 7.2. Personalized Nutritional Interventions

In a randomized crossover clinical trial, Link et al. investigated the impact of nutrition, specifically ketogenic and vegan diets, on both the peripheral immune response and the body’s microbiome composition. Twenty participants were divided into two groups, groups A and B, and remained on-site for the entire 4-week period at the Metabolic Clinical Research Unit at the NIH Clinical Center to ensure good control of the dietary interventions. Each group comprised both males and females and boasted a diversity of ethnicity, race, age, and body mass index. Each participant ate a keto or vegan diet for two weeks, then switched to a keto or vegan diet for another two successive weeks. The vegan diet was a low-fat diet with 10% fat and 75% carbohydrates, while the keto diet was low in carbohydrates and rich in fats: 75.8% fat and 10.0% carbohydrate. Using a multi-omics approach comprising metagenomic, metabolomic, proteomic, and transcriptomic datasets, Link et al. found that a vegan diet had a substantial impact on the innate immune system and antiviral immunity, while the ketogenic diet had a significant impact on adaptive immunity [83]. 

Link and colleagues suggest that it may be possible to personally tailor one’s diet towards disease prevention and treatment since the immune system responds swiftly to nutritional interventions [83]. This is the first type of multi-omics study to investigate the impact of vegan and keto diets in humans. It is ushering in a whole new era in precision nutrition and a deeper understanding of diet-based therapeutics for the treatment and mitigation of disease. This was a small study with only 10 participants in the treatment group. It would be beneficial to pilot these interventions for LC treatments.

Shedding light on nutrition and its impact on restoring the gut microbiota is beneficial since short-chain fatty acids released from dietary fibers by the gut microbes play a critical role in modulating the immune system and overall health maintenance. Nutritional supplements such as antioxidants and essential fatty acids could alleviate and manage LC symptoms [84]. Deficiencies in Vitamins B and C, coenzyme Q, sodium, magnesium, zinc, folic acid, tryptophan, and essential fatty acids contribute to the progression and severity of CFS. Thus, a balanced healthy diet with sufficient supplements and antioxidants could help in alleviating LC fatigue syndrome [85]. 

### 7.3. Public Health Implications

LC is already being addressed as a significant public health crisis. Despite many studies, trials, and interventions, the underlying mechanisms behind the condition are still unknown. As there is no known way of addressing the root cause, current treatments are limited to managing individual symptoms with a focus on recovering a good quality of life despite the condition. Due to the chronic nature of LC, as well as the sheer number of symptoms, current treatments can also seem insufficient, inconvenient, and even ineffective for many individuals. Certain cases of LC are debilitating enough to be individually categorized as legal disabilities (U.S. Department of Health and Human Services), greatly hampering productivity in the workplace and leading to negative economic outcomes for individuals and their families. The fact that the number and severity of LC symptoms are indeterminable regardless of the nature of prior COVID-19 infection and that symptoms can persist and recur over several years, make LC an utmost priority for public health worldwide. Given current knowledge of the condition, measures should be taken to improve the quality and capacity of LC symptom interventions and relief until information about the underlying mechanisms is available.

## 8. Conclusions

Researching the underlying mechanisms of LC is critical from a public health perspective. While helping patients cope with symptoms and recover is crucial, it should be treated as a temporary measure, not a solution. Given the multiorgan involvement of LC symptoms, the true nature of the condition cannot be elucidated linearly and may require significant lengths to understand the variability of symptoms on a case-by-case basis. LC characterization and research are still in their early stages, and more is yet to be discovered [86]. Given the complexity of LC and the sheer millions impacted by LC, managing and alleviating LC symptoms should be a top priority and imperative for public health.

## Figures and Tables

**Table 1:** Clusters representing subtypes of LC clinical manifestations and diagnoses [51].

Neurological Cluster	Comorbidity Cluster	Gastrointestinal Cluster	Upper Respiratory Cluster	Cardiopulmonary Cluster
Anxiety Disorder	Blood Chemistry Abnormalities	Abdominal Pain	Acute Pharyngitis	Chest Pain
Chronic Fatigue Syndrome	Essential Hypertension	Constipation	Acute Upper Respiratory Infection	Chronic Cough
Chronic Pain	Gastroesophageal Reflux Disease without Esophagitis	Diarrhea	Allergic Rhinitis	Cough
Depressive Disorder	Hyperlipidemia	Disorder Following Viral Disease	Chronic Cough	Dyspnea
Dizziness	Morbid Obesity	Fever	Cough	Palpitations
Fatigue	Obesity	Multisystem Inflammatory Syndrome	Nasal Congestion	Tachycardia Cough
Findings Related to Attentiveness	Obstructive Sleep Apnea Syndrome	Nausea And Vomiting	Sensory Disorder of Smell and/or Taste	
Generalized Anxiety Disorder	Type 2 Diabetes without Complications	Viral Disease	Uncomplicated Asthma	
Headache	Vitamin D Deficiency	Vomiting		
Insomnia				
Joint Pain				
Muscle Pain				
Nausea				
Sensory Disorder of Smell and/or Taste				

**Table 2:** Selected drugs in clinal trials for LC treatment.

Clinical Trial Identifier	Drug	Target
NCT04448119 ^1^	Favipiravir/Avigan	RNA-dependent RNA polymerase inhibitor
NCT05595369 ^2^	Paxlovid	Protease Inhibitor
NCT04978259 ^3^	Remdesivir	RNA-dependent RNA polymerase inhibitor
NCT05220280 ^4^	Infliximab/Remicade	TNF alpha Inhibitor
NCT05220280 ^5^	Imatinib/Glivec	Kinase Inhibitor
NCT05513560 ^6^	Ibudilast	Phosphodiesterase Inhibitor

^1^ [60]; ^2^ [61]; ^3^ [62]; ^4^ [63]; ^5^ [63]; ^6^ [64].
